# Supplementary material for: piR-bmo-796514 facilitates the proliferation of exogenous DNA virus (baculovirus) by targeting the host E3 ubiquitin ligase RNF181
Source: PLoS Pathog. 2026 Jan 6;22(1):e1013848. doi: 10.1371/journal.ppat.1013848 (PMC12799189; doi:10.1371/journal.ppat.1013848)
Supplement: S1 Table — (DOCX) [file ppat.1013848.s006.docx]

**S1 Table:**

| **Sequences name** | **Sequence of Oligonucleotide (5’-3’)** |
| --- | --- |
| piR-bmo-796514-mimics | sense: CAAUUUUCGCCCUCGAUGGGGAUC |
|  | antisense: UCCCCAUCGAGGGCGAAAAUUGUU |
| piRNA-mimics-NC | sense: UUCUCCGAACGUGUCACGUTT |
|  | antisense: ACGUGACACGUUCGGAGAATT |
| piR-bmo-796514-agomir | sense: CAAUUUUCGCCCUCGAUGGGGAUC |
|  | antisense: UCCCCAUCGAGGGCGAAAAUUGUU |
| piRNA-agomir-NC | sense: UUCUCCGAACGUGUCACGUTT |
|  | antisense: ACGUGACACGUUCGGAGAATT |
| piR-bmo-796514-inhibitor | GAUCCCCAUCGAGGGCGAAAAUUG |
| piRNA-inhibitor-NC | CAGUACUUUUGUGUAGUACAA |
| piR-bmo-796514-antagomir | GAUCCCCAUCGAGGGCGAAAAUUG |
| piRNA-antagomir-NC | CAGUACUUUUGUGUAGUACAA |
| pIEX-RNF181-V5 | F:CATCGTTAACACGTCAAGAGCTCATGGCTGGTTATTTTGAAGA |
|  | R:GAACCGGTACCGTCGACCTACGTAGAATCGAGACCGAGGAGAGGGTTAGGGATAGGCTTACCACTAAACATTGAATTGTG |
| pIEX-Integrin α2b-like-His | F:CAACCAAGTGACCATGGGCATGTTTGGCCTAAGAA |
|  | R:ATTGGGATCCGCGAGCTCTTACGTAGAATCGAGACCGAGGAGAGGGTTAGGGATAGGCTTACCATCTTCCAACGATGTTA |
| pGL3-RNF181-WT | F:ATTTCTCTATCGATAGGTACCGAGCTCATGGCTGGTTATTTTGAAGAAA |
|  | R:CAGTACCGGAATGCCAAGCTTTTTGGGAATATTTTAATTTA |
| pGL3-RNF181-Mut | F: ATGAGATGCCTACTGATGACGGACGCGATGAAGCCT |
|  | R: AGGCTTCATCGCGTCCGTCATCAGTAGGCATCTCAT |
| pIEX-Integrin α2b-like-NVR-His-Mut | F:CGAAAATTTATTGGTGGCCGCGGCTAACGATGGGCACGCA |
|  | R:TGCGTGCCCATCGTTAGCCGCGGCCACCAATAAATTTTCG |
| pIEX-Integrin α2b-like-VAL-His-Mut | F:CGAGAACTATTGTGGGCGCCAAGGAAGGCGCCCGTTTC |
|  | R:GAAACGGGCGCCTTCCTTGGCGCCCACAATAGTTCTCG |
| pIEX-Integrin α2b-like-ASN-His-Mut | F:AACATATTACACTGAGGCCCCATTAAAGGACAAGAAA |
|  | R:TTTCTTGTCCTTTAATGGGGCCTCAGTGTAATATGTT |
| pIEX-Integrin α2b-like-LYS-His-Mut | F:CATTAAAGGACAAGGCCCCGATGATTGTACTGAT |
|  | R:ATCAGTACAATCATCGGGGCCTTGTCCTTTAATG |
| qPCR-piR-bmo-796514 | F:AACAATCAATTTTCGCCCTCGA |
| qPCR-Stem-loop | R:CTCAACTGGTGTCGTGGA |
| qPCR-u6 | F: CTCGCTTCGGCAGCACA |
|  | R: AACGCTTCACGAATTTGCGT |
| qPCR-RNF181 | F: GGGTGATGGGGAACAACCAA |
|  | R: GAGGTAAGCTTGGCCACTCC |
| qPCR-Integrin α2b-like | F: TGGAAGTTAACGGGGCACAG |
|  | R: CATTTCTCTCGGCCTCCCTC |
| qPCR-tif4a | F: GAATGGACCCTGGGACACTT |
|  | R: CTGACTGGGCTTGAGCGATA |
| qPCR-vp39 | F: CTAATGCCCGTGGGTATGG |
|  | R: TTGATGAGGTGGCTGTTGC |
| qPCR-gp41 | F: ATGTTGATGTGCGGAAAGC |
|  | R: GTGGCGGAATCGGTGA |
| dsRNA-RNF181 | F:TAATACGACTCACTATAGGGGGTTGGAGAGAATTGGGTGA |
|  | R: TAATACGACTCACTATAGGGTCATGTCTACAAAACGGGCA |
| dsRNA-Integrin α2b-like | F: TAATACGACTCACTATAGGGCGCCACGGTATACGAAGATT |
|  | R:TAATACGACTCACTATAGGGGTTCGAAACACTGGCGTTCT |
| dsRNA-DsRed | F:TAATACGACTCACTATAGGGGAAGCTGAAGGTGACCAAGG |
|  | R: TAATACGACTCACTATAGGGTGGTGTAGTCCTCGTTGTGG |

The underline represents restriction sites.

The wavy lines indicate the sequence of mutations.

The sequences of mimics and agomir are the same, and the sequences of inhibitor and antagomir are identical; however, they differ in their modifications (Detailed information is provided in the main text).
